# Study protocol for a type III hybrid effectiveness-implementation trial to evaluate scaling interoperable clinical decision support for patient-centered chronic pain management in primary care

**DOI:** 10.1186/s13012-022-01217-4

**Published:** 2022-07-15

**Authors:** Ramzi G. Salloum, Lori Bilello, Jiang Bian, Julie Diiulio, Laura Gonzalez Paz, Matthew J. Gurka, Maria Gutierrez, Robert W. Hurley, Ross E. Jones, Francisco Martinez-Wittinghan, Laura Marcial, Ghania Masri, Cara McDonnell, Laura G. Militello, François Modave, Khoa Nguyen, Bryn Rhodes, Kendra Siler, David Willis, Christopher A. Harle

**Affiliations:** 1 Department of Health Outcomes and Biomedical Informatics, University of Florida College of Medicine, 2004 Mowry Road, Gainesville, FL 32610 USA; 2 Department of Medicine, University of Florida College of Medicine, Jacksonville, FL USA; 3 Applied Decision Science, Cincinnati, OH USA; 4 Department of Anesthesiology, Wake Forest University School of Medicine, Winston-Salem, NC USA; 5 Department of Community Health and Family Medicine, University of Florida College of Medicine, Jacksonville, FL USA; 6 RTI International, Research Triangle Park, NC USA; 7 Department of Pharmacotherapy and Translational Research, University of Florida College of Pharmacy, Gainesville, FL USA; 8 Alphora, Orem, UT USA; 9 CommunityHealth IT, Kennedy Space Center, Merritt Island, FL USA

## Abstract

**Background:**

The US continues to face public health crises related to both chronic pain and opioid overdoses. Thirty percent of Americans suffer from chronic noncancer pain at an estimated yearly cost of over $600 billion. Most patients with chronic pain turn to primary care clinicians who must choose from myriad treatment options based on relative risks and benefits, patient history, available resources, symptoms, and goals. Recently, with attention to opioid-related risks, prescribing has declined. However, clinical experts have countered with concerns that some patients for whom opioid-related benefits outweigh risks may be inappropriately discontinued from opioids. Unfortunately, primary care clinicians lack usable tools to help them partner with their patients in choosing pain treatment options that best balance risks and benefits in the context of patient history, resources, symptoms, and goals. Thus, primary care clinicians and patients would benefit from patient-centered clinical decision support (CDS) for this shared decision-making process.

**Methods:**

The objective of this 3-year project is to study the adaptation and implementation of an existing interoperable CDS tool for pain treatment shared decision making, with tailored implementation support, in new clinical settings in the OneFlorida Clinical Research Consortium. Our central hypothesis is that tailored implementation support will increase CDS adoption and shared decision making. We further hypothesize that increases in shared decision making will lead to improved patient outcomes, specifically pain and physical function. The CDS implementation will be guided by the Exploration, Preparation, Implementation, Sustainment (EPIS) framework. The evaluation will be organized by the Reach, Effectiveness, Adoption, Implementation, and Maintenance (RE-AIM) framework. We will adapt and tailor PainManager, an open source interoperable CDS tool, for implementation in primary care clinics affiliated with the OneFlorida Clinical Research Consortium. We will evaluate the effect of tailored implementation support on PainManager’s adoption for pain treatment shared decision making. This evaluation will establish the feasibility and obtain preliminary data in preparation for a multi-site pragmatic trial targeting the effectiveness of PainManager and tailored implementation support on shared decision making and patient-reported pain and physical function.

**Discussion:**

This research will generate evidence on strategies for implementing interoperable CDS in new clinical settings across different types of electronic health records (EHRs). The study will also inform tailored implementation strategies to be further tested in a subsequent hybrid effectiveness-implementation trial. Together, these efforts will lead to important new technology and evidence that patients, clinicians, and health systems can use to improve care for millions of Americans who suffer from pain and other chronic conditions.

**Trial registration:**

ClinicalTrials.gov, NCT05256394, Registered 25 February 2022.

**Supplementary Information:**

The online version contains supplementary material available at 10.1186/s13012-022-01217-4.

Contributions to the literature

- This study will adapt and scale existing open-source interoperable CDS for patient-centered chronic pain care.
- The research will generate evidence on strategies for implementing interoperable CDS in new clinical settings across different types of EHRs.
- The study will also inform tailored implementation strategies to be further tested in a subsequent hybrid effectiveness-implementation trial.
- Together, these efforts will lead to important new technology and evidence that patients, clinicians, and health systems can use to improve care for millions of Americans who suffer from pain and other chronic conditions.

## Background

Over the past decade, widespread electronic health record (EHR) adoption has provided a technology platform on which clinical decision support (CDS) could be delivered more broadly and within workflows [1–3]. At the same time, Fast Healthcare Interoperability Resources (FHIR) has emerged as a promising standard for health information exchange on which CDS systems can be integrated within and across organizations. Also, medical informaticians developed the Substitutable Medical Applications, Reusable Technologies (SMART) on FHIR application programming interface (API), developer tools, and app gallery for disseminating CDS [4, 5]. Complementing these technological advances, the 21^st^ Century Cures Act supports the promotion of interoperable CDS by requiring federally certified EHRs to provide patients API level access to their EHR data [6]. As these standards mature, there is a critical need to evaluate interoperable CDS implementation and impact on care quality and patient outcomes.

While recent technology standards and policies are promising, health information technology (IT) implementations often fail [7, 8]. Usable health IT must align with the sociotechnical environment, including organizational structures, clinical workflows, and user information needs and decision-making processes [9–11]. Therefore, implementation science is critically needed to generate evidence on how to scale interoperable CDS.

For this project, we chose the important clinical domain of chronic noncancer pain care in primary care settings. Chronic pain affects an estimated 50 to 100 million Americans at an estimated cost of more than $600 billion annually [12, 13]. Over the last three decades, the US healthcare system dramatically increased opioid prescribing for chronic noncancer pain which contributed to an epidemic of opioid use disorder and overdose deaths [14–19]. Since its US peak in 2012, opioid prescribing and dispensing have declined significantly [20–23]. Still, more than 142 million opioid prescriptions were dispensed in 2020 [23]. Consequently, chronic pain remains widespread, patients are suffering, and clinicians are struggling to choose safe and effective treatments. Using CDS for shared decision making, providers and patients can collaboratively assess risks, benefits, patients’ symptoms and goals, available resources, and choose treatments that lead to improved pain and function.

Some of the earliest efforts to implement and evaluate CDS for chronic noncancer pain care identified barriers to adoption and use, including information overload, insufficient time during routine visits, and lack of clinically actionable guidance [24–27]. Subsequent research focused on user-centered design of CDS that is aligned with providers’ information needs, common clinical tasks, and workflow constraints [28]. As attention to the opioid crisis increased, more CDS tools emerged [29], including several that used interoperable technologies, namely PainManager [30–32]. However, to date, most research on CDS for chronic pain lacks rigorous implementation and effectiveness evaluations [29]. The proposed project will help close evidence gaps, improve knowledge on scaling interoperable CDS to new organizations, and improve knowledge on how to design multi-site implementation and effectiveness trials of interoperable CDS.

## Methods/design

This protocol adheres to the Standards for Reporting Implementation Studies (StaRI) Statement (Additional file 1) [33].

### Regulatory approvals

This study was registered on ClinicalTrials.gov on February 25, 2022 (NCT05256394). The University of Florida Institutional Review Board (IRB) serves as the single IRB (sIRB). The study was approved on October 27, 2021 (IRB#202101931).

### Conceptual framework and rationale for approach

**Fig. 1:**
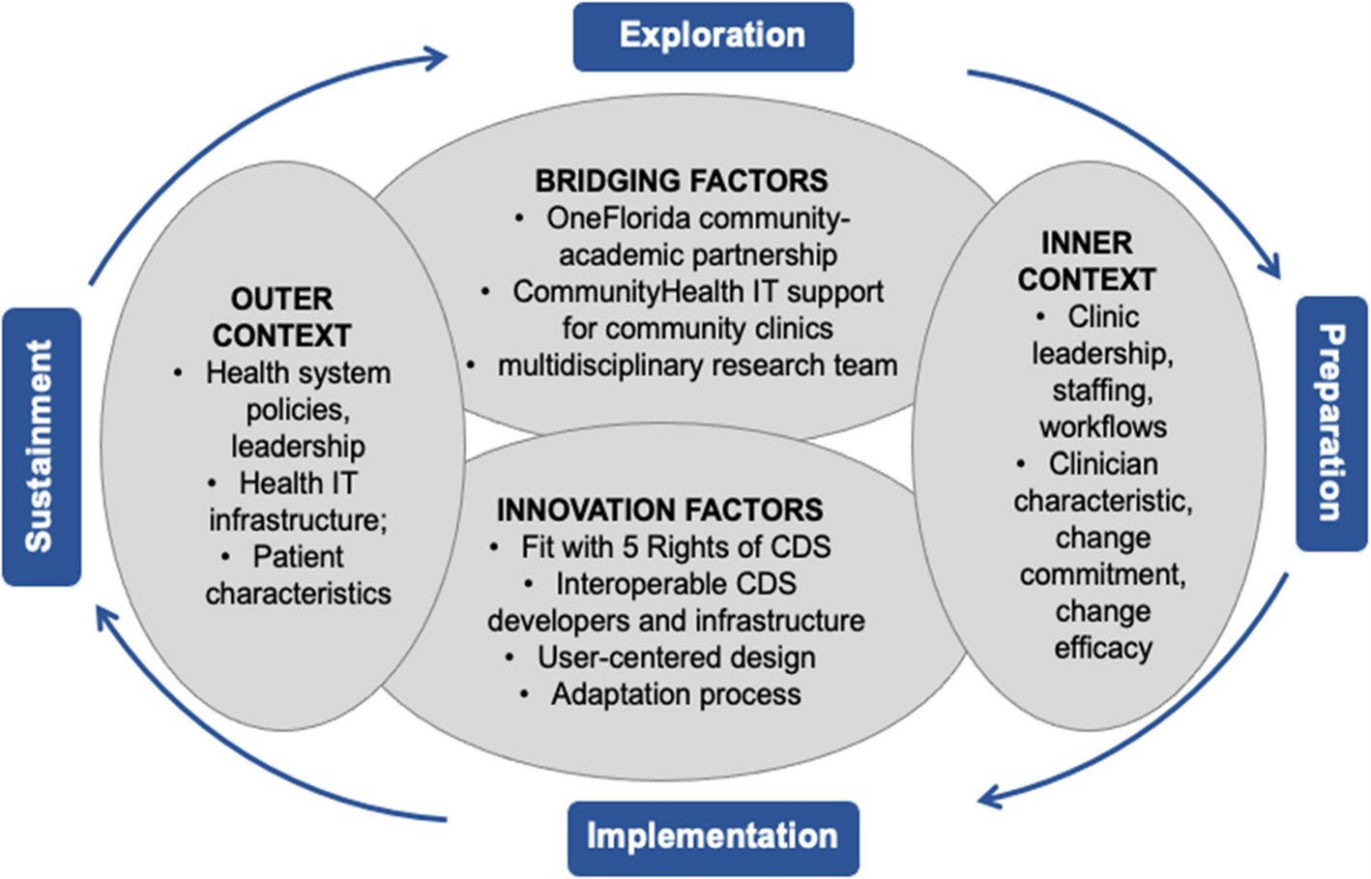
EPIS framework applied to interoperable clinical decision support for chronic pain management

The Exploration, Preparation, Implementation, Sustainment (EPIS) framework [34] serves as the overarching framework (Fig. 1) for this implementation. Application of EPIS will optimize the relevance of our findings for generalizability and scalability. EPIS describes four phases of implementation for evidence-based interventions and the factors that influence the implementation process and outcomes [34, 35]. The “Exploration” phase begins when stakeholders become aware of a public health or clinical need and thus includes our team’s prior work [27, 28, 36–42], prior interoperable CDS development for chronic pain [31, 43], and the development of this protocol. The “Preparation” phase objectives are to identify barriers and facilitators to implementation, assess need for adaptation, and develop an implementation plan. In this study, the preparation will involve translating PainManager adaptations (Aim 1) and pre-implementation evaluation data (Aim 2) to a plan for integrating PainManager in the local EHR for rollout in primary care clinics. The “Implementation” phase will involve instantiating PainManager in the local EHR environment, including final technical and workflow integrations, CDS go-live in routine clinical practice, and monitoring and evaluating CDS adoption and effectiveness (Aims 2 and 3). In this study, “Sustainment” involves maintaining and adapting the structures, processes, and supports needed to ensure PainManager use and effectiveness over time. EPIS will also guide our understanding of the multi-level factors that could influence implementation, organized in four inter-related domains: *inner context*, *outer context*, *bridging factors*, and *innovation factors*. In this study, PainManager’s alignment with the CDS 5 Rights (right information, right person, right intervention format, right channel, and right time in workflow) represents a key innovation factor and will be assessed throughout the study [44]. Finally, specific evaluation measures will span each of the constructs in the Reach, Effectiveness, Adoption, Implementation, Maintenance (RE-AIM) framework [45]. Our central hypothesis is that tailored CDS implementation support will increase CDS adoption and shared decision making. We further hypothesize that increases in shared decision making will lead to improved patient outcomes for managing pain and improving physical function (i.e., clinical effectiveness).

### Study sites and populations

Our decision support design and evaluation will be conducted with a stakeholder group drawn from primary care practices affiliated with the OneFlorida Clinical Research Consortium. OneFlorida is a collaborative statewide clinical research network funded by the Patient-Centered Outcomes Research Institute (PCORI) that includes 12 healthcare delivery and payer partners providing care for 60% of all Floridians through 4100 physician providers, 1240 clinic/practice settings, and 22 hospitals with a catchment area covering all 67 Florida counties. We will focus on clinics affiliated with two OneFlorida partners, University of Florida (UF) Health and CommunityHealth IT (CommHIT).

### Intervention—existing interoperable CDS for pain management

**Fig. 2:**
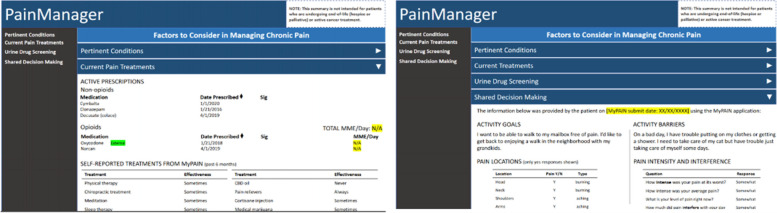
PainManager interface. Left: current pain treatments; Right: patient-reported goals and barriers from MyPAIN. CQF. Clinical Decision Support for Chronic Pain Management and Shared Decision-Making IG. Retrieved 15 April 2022 from: https://build.fhir.org/ig/cqframework/cds4cpm/

We will adapt, implement, and evaluate an existing clinician-facing CDS tool, PainManager, and a connected patient-facing system, MyPAIN. Each tool was developed with funding support from AHRQ and is freely available via public GitHub repositories with an associated implementation guide [46, 47]. Together, these tools disseminate evidence-based patient-centered outcomes research (PCOR) findings related to chronic pain*.* Note, in this protocol, we often use the term “PainManager” as shorthand to refer to both PainManager and MyPAIN, differentiating only as needed for clarity. PainManager is an EHR dashboard-like application that displays key information needed by clinicians to engage in shared pain treatment decision making with patients (Fig. 2). The dashboard displays relevant patient conditions (i.e., diagnoses), current pain treatments, information relevant to opioid risk (e.g., morphine milligram equivalent [MME] daily dose, urine drug screen results), and patient-reported information. Patient-reported information is collected via MyPAIN. Together, MyPAIN and PainManager collect treatment decision-relevant information from patients and the EHR and display the information to aid patient-provider discussions and choices of pain treatments that balance risks and benefits in the context of past treatments, symptoms, and patient goals.

The existing CDS architecture triggers a patient portal message when a patient with chronic noncancer pain schedules a clinic visit. From the message, patients can launch MyPAIN. Patient-reported information from MyPAIN is communicated to the EHR via FHIR resources, which are also used to display patient-reported information in the PainManager dashboard. Providers can then invoke PainManager for their EHR (e.g., Epic) and engage patients in shared decision making. PainManager uses FHIR version 4 specifications to read information from an EHR and MyPAIN uses FHIR version 4 specifications to write information to an EHR. Because EHR vendors’ APIs have different and evolving levels of support for FHIR resources, PainManager and MyPAIN were designed with a “FHIR Façade” layer to support data exchange with Epic at current implementation sites. In the proposed project, the CDS will be adapted as needed to fit with UF Health’s local data structures and current FHIR API support and to fit with local clinician and patient needs in the context of the CDS 5 Rights.

### Overview of methods

**Fig. 3:**
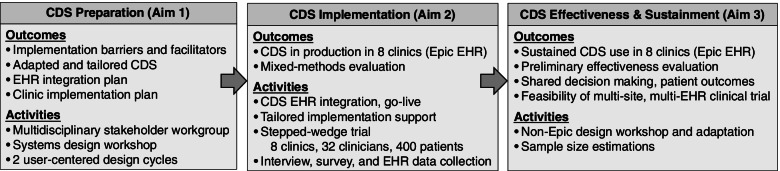
Overview of study activities and outcomes

Figure 3 overviews the primary activities and outcomes involved in all three study aims. We will convene a multi-disciplinary stakeholder workgroup and system design workshops and 2 user-centered design cycles to adapt and tailor PainManager to local context (Aim 1). Then, we will conduct a mixed-methods, pragmatic, stepped-wedge, cluster-randomized trial, with a hybrid type 3 implementation-effectiveness design to evaluate (1) the impact of tailored implementation support on PainManager adoption by primary care clinicians as measured in the EHR (Aim 2), while also (2) considering pain management shared decision making and the health impacts of this adoption (Aim 3). Pre- and post-implementation clinician interviews and surveys will be conducted to inform workflow integration and tailoring to each clinic and to evaluate the implementation process. In parallel with the trial, we will conduct a technical need assessment and preliminary adaptation of PainManager for use in two additional clinics that use non-Epic EHRs. This activity will demonstrate feasibility of broader CDS scaling to support a full-scale trial across multiple OneFlorida Consortium health systems.

### Approach for aim 1

#### Participant sample and recruitment

We will recruit approximately 9 workgroup members to participate in the system design workshop and to guide the study team in the process of adapting and tailoring PainManager to UF Health. For the workgroup, we will recruit primary care clinicians, clinical IT interface analysts, citizen scientists (patients with chronic pain), and pain specialist physicians.

**Fig. 4:**
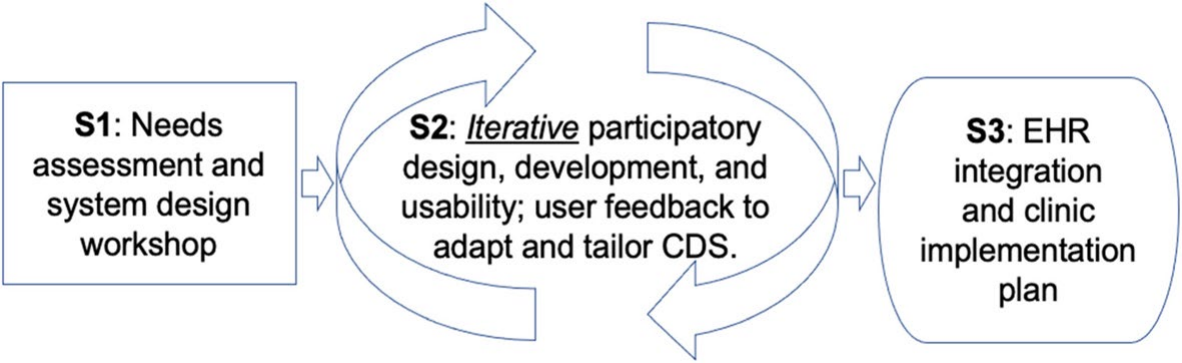
User-centered design process for pain management clinical decision support

For the participatory design usability studies (Fig. 4), we will recruit both care team members and patient participants (*n*=48 total). For care team member participants, we will recruit primary care providers who care for patients with chronic pain, including physicians, nurse practitioners, and physician assistants. We will also include other primary care team staff who support workflows related to pain care (e.g., medical assistants, nurses, social workers). We will recruit clinician and care team member participants via emails, phone calls, or in-person and facilitated by clinical leadership. We will target patient participants with a range of chronic noncancer musculoskeletal pain conditions, including those currently being treated with opioids and those not currently being treated with opioids.

#### System design workshop procedure (S1)

First, we will conduct a full-day system design workshop with the workgroup, our study team, and software developers. We will present a summary of the functionality of the existing PainManager CDS application, and our team’s prior work to design user centered CDS for pain treatment decision making. Next, through facilitated discussion, we will establish group consensus on potential design adaptations to PainManager’s interface and functionality that enhance local usability, fit with the CDS 5 Rights, and provide clinical usefulness for shared decision making.

#### Iterative participatory design, development, and usability procedures (S2)

We will conduct two 3-month cycles of iterative participatory design activities. These cycles will rapidly produce revised system requirements, high-fidelity prototypes, and progress the existing PainManager (and MyPAIN) application for implementation at UF Health. In each cycle, the full workgroup will meet monthly, and sub-teams will meet weekly with software developers, user interface visual design staff, and clinical IT integration staff from each site. In months 1–2 of each cycle, the workgroup will follow a rapid iterative software development process to design the system’s updated visual interface and specifications for data exchange and analytic processing.

In month 3 of each cycle, we will conduct 90-min think-aloud interviews with patients and care team members to inform PainManager usability. With consent, we will capture audio and screen recordings to collect participant feedback and interactions with both patient-facing and clinician-facing components of the CDS. We will assess usability of the user interface with the System Usability Scale (SUS) [48]. Using a *think-aloud* protocol [49], participants will be asked to individually interact with the prototypes and to verbalize their experience. Usability issues will be encoded using themes derived from a *thematic analysis* of data captured during the interviews and mapped to *usability heuristics* defined in *Gerhardt-Powal’s* cognitive engineering principles on usability violations [50]. The interview results will be used to glean user preferences and barriers to using the system and specifically to support shared decision making in pain treatment choices. A commonly recommended sample size for SUS is 12. Therefore, we will recruit 12 care team members and 12 patient participants in each cycle to evaluate clinician- and patient-facing components [51]. We will recruit for diversity in gender and role (care team participants) and for gender and pain condition (patient participants). Our goal is to achieve SUS>68, the average SUS score from more than 300 web applications [52].

The system design workshop (S1) and all development sessions and think-aloud interviews (S2) will be recorded and attended by a research assistant with biomedical informatics expertise. The research assistant will be dedicated to notetaking and observing the software adaptation and tailoring development process. These observations, along with developer notes and software documentation created in the process, will be transcribed and combined to provide a diverse set of qualitative data. In parallel to the software development work, we will thematically analyze these data to produce new scientific findings on barriers and facilitators to adapting interoperable CDS to new organizations and conforming with the CDS 5 Rights. A subgroup of study team members will begin by independently reading and open coding the transcripts, notes, and documentation. In an iterative process, we will develop and finalize an analysis codebook, code all data, and use a process of upward abstract to identify general themes.

This aim will produce an adapted and tailored version of PainManager for implementation and evaluation in primary care practices at UF Health (S3). All updates to PainManager code and documentation will be disseminated via GitHub. This aim will also produce presentations and publications that describe qualitative findings produced from interviews, notes, and other documents generated during the system design process.

### Approach for aim 2

#### Recruitment and randomization of practices: stepped-wedge trial

**Fig. 5:**
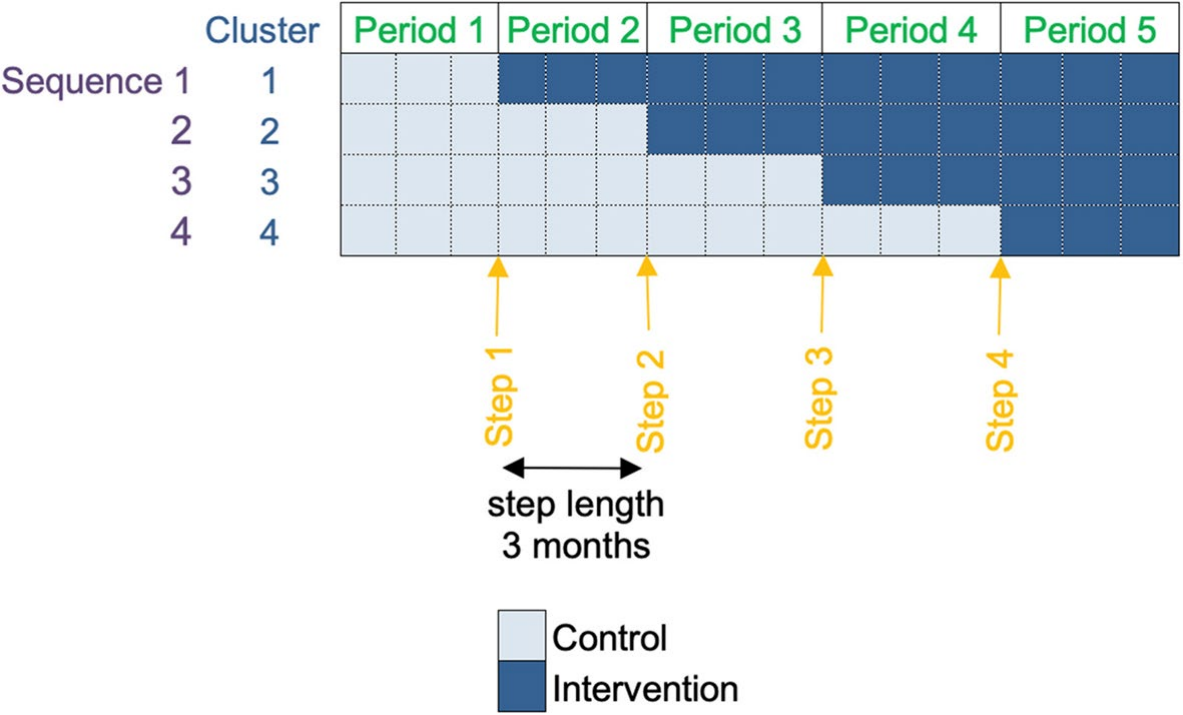
Study diagram with key terminology as recommended by the Consolidated Standards of Reporting Trials (CONSORT) extension for stepped wedge randomized controlled trials. Note: each cluster include two primary care clinics from the University of Florida Health System in Jacksonville, FL

Eight clinics will be recruited and randomized to the staggered receipt of tailored implementation support. Participating clinics will be asked to identify a clinician champion and/or an operation champion to take part in implementation support activities. The clinics will receive a description of the tasks involved with each role and may select staff for these roles as they deem appropriate. All study clinics will begin the trial with PainManager integrated and available in their EHR. Then, the focal intervention (i.e., tailored CDS implementation support) will be rolled out, with randomization staggering the start. In summary, we are proposing a complete, open-cohort stepped-wedge cluster-randomized design [53] with 5 time periods (including the baseline), with each period spanning 3 months and 2 clinics switching from control to intervention at each of the 4 steps (for a total study duration of 15 months). Figure 5 shows a study diagram with key terminology as recommended by the Consolidated Standards of Reporting Trials (CONSORT) extension for stepped-wedge cluster randomized trials [54].

#### Patient eligibility

Qualifying patients will be adult (≥ 18 years) primary care patients whose EHRs indicate chronic noncancer musculoskeletal pain and a recent history of opioid use. We plan to identify such patients, for whom PainManager use will be triggered, based on the following criteria in the past 12 months: (1) 2+ visits to a primary care clinic, (2) 3+ instances of a musculoskeletal pain diagnosis separated by at least 30 days, (3) no history of a cancer diagnosis in the past three years, and (4) 45+ days’ supply of an opioid prescription.

#### Implementation support components

We will provide clinics with a tailored implementation support package informed by Aim 1 findings and pre-implementation data collection. Consistent with the stepped-wedge design, intensive implementation support will be provided to two clinics at once by a multidisciplinary team. The implementation support team activities will be co-led by the principal investigators and physician co-investigators and will have technical, clinical, and research staff support. In the first 3 months, we expect the support team will spend 5–7 h per clinic per week, with support time decreasing significantly thereafter. Per clinic in the first 3 months, we estimate 2 h/week in office hours, 1 h/week in clinical champion check-ins, 1–2 h/week responding to emails, and 1–2 h/week in on-demand phone/computer-based support sessions.

Within these interactions, we will deliver tailored support using strategies selected (1) for their demonstrated effectiveness at supporting practice change [55, 56], (2) experience from our prior work, and (3) potential scalability. These strategies include staff training, technical assistance, audit and feedback, goal identification, leadership engagement, practice coaching, peer-to-peer learning, orientation materials, and implementation guides/CDS tip sheets. The approach is based on evidence that practice change is best supported by a combination of implementation strategies, positing that CDS tools are more likely to be adopted with guidance on use. We expect much of the implementation support will be conducted one-on-one. Implementation support team members will be available on call to meet users’ time preferences.

##### Office hours

An implementation support team coach will hold in-person “office hours” at each clinic. The coach will be available for individualized support as clinicians are available (e.g., over the lunch hour or between patients). The coach will also be prepared to focus on one aspect of CDS use, determined by clinic request or the coach’s knowledge of the clinics’ progress. To support individual clinicians’ limited availability and differing schedules, the coach will spend two hours on site.

##### Peer support

To support peer-to-peer learning, we will encourage clinicians who have made progress with use to share experiences directly with their peers. However, we will also capture success stories in brief tip sheet handouts and/or brief video demos/testimonials, which can be delivered by study staff directly to other participating clinicians during office hours.

##### Weekly coaching call

A member of the implementation support team will meet with each clinic’s champion by phone for one hour to review the clinic’s CDS adoption (monitored via EHR logs), ask about barriers/facilitators to CDS adoption, and otherwise help as needed.

##### Email questions

Study clinics will be encouraged to email the implementation support team with questions. The support team will respond within one workday. We recognize that many clinicians may not proactively reach out for support via email. Study staff will send weekly check-in emails with news and CDS tips to help prompt questions.

##### Phone calls/support sessions

Study staff will be available for on-demand phone calls and computer-based support sessions and will respond within 24 h.

#### Clinician surveys and in-depth interviews

Pre- and post-implementation, we will invite all providers (*n*=32) and non-provider clinical care team members, e.g., social workers, nurses, medical assistants (*n*=40) to complete a survey. We will assess participant characteristics (i.e., age, race, ethnicity, gender, experience, training/specialty), change commitment and change efficacy with respect to CDS implementation and shared decision making, and acceptability and appropriateness of PainManager features and functionality in the context of the CDS 5 Rights. To maximize response rates, we will administer surveys using the REDCap electronic survey system and via paper surveys distributed to clinics. Surveys will be piloted before administration and take no more than 15 min to complete. To complement and expand on the survey data, each survey administration will be followed by in-depth interviews with 2 providers at each clinic (medical director and one other, *n*=16). Findings from the surveys will inform adaptations to the interview guide specific to the context of each clinic. The interviews will elicit attitudes towards and experiences with pain care and potential barriers and facilitators to implementing and sustaining CDS use, including outer setting, inner setting, and intervention-level factors. Interviews will last 30–45 min, be audio-recorded, and transcribed verbatim.

#### Measures

Outcome measures will include data from clinic EHRs, surveys, and interviews (Table 1). Each clinic will provide data for both control and intervention conditions. The *primary* outcome, CDS *adoption* in clinical encounters by qualifying patients, will be assessed from EHR activity logs. The rate of qualifying encounters in which clinician CDS use is documented will be used to measure *reach.*

**Table 1:**
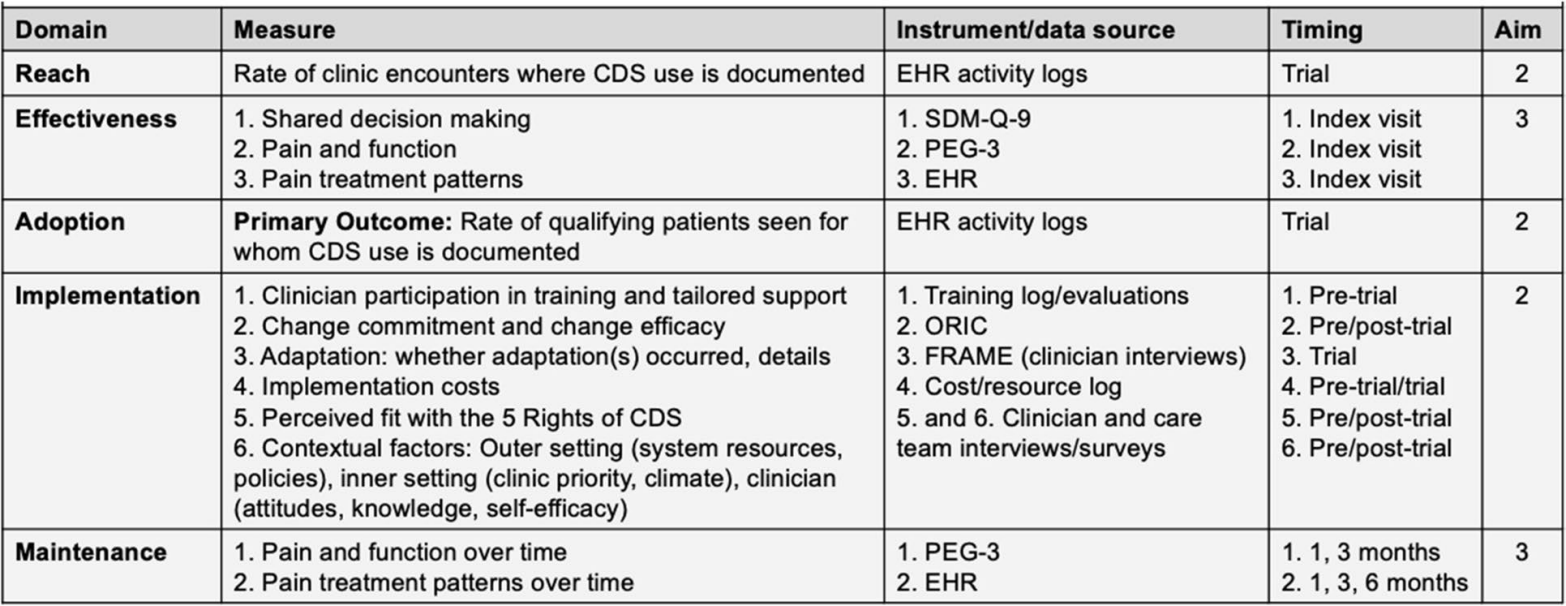
Outcome measures, data sources, and timing of data collection

We will evaluate the *implementation* process using several measures. First, we will assess *clinician training attendance and quality*, as well as participation in implementation support activities, as measures of fidelity [57].

Second, the clinician surveys will include the *organization readiness for implementing change* (ORIC) instrument [58] to assess *change commitment* (i.e., clinicians’ shared resolve to use PainManager in shared decision making) and *change efficacy* (i.e., clinicians’ shared belief in their collective capability to use PainManager in shared decision making). ORIC is a 12-item instrument used to determine how well individuals in an organization feel they can implement the change in processes required by an intervention. Each item includes a Likert scale from 1 (“disagree”) to 5 (“agree”).

Third, we will use prospective activity-based costing to evaluate *implementation costs*, following AHRQ’s Practical Guide for Estimating the Costs of Primary Care Transformation [59]. We will collect data on estimates of the time spent using the CDS and delivering shared decision making using the clinician surveys and electronic logs. We will assess costs to deliver the intervention, including personnel resources, training materials, and technology costs. To maximize the generalizability of results and minimize the potential that performance sites are not representative of typical salary structures, cost components (e.g., wages, travel costs) will be based on national average estimates. We will access national public databases to determine cost estimates for variables such as travel expenses and salaries for implementation activities. Intervention costs will exclude research costs.

Fourth, in-depth interviews with providers will first assess the perceived fit of PainManager with the CDS 5 Rights and elicit reasons in case of suboptimal fit. Interviews will also assess contextual factors that include multilevel barriers and facilitators to implementation and sustainment, organized according to the EPIS framework [34] into outer setting (e.g., system resources), inner setting (e.g., care climate), and provider (e.g., attitudes towards the intervention).

#### Sample size considerations

Statistical power considerations focus on our primary measure, CDS adoption, comparing rates of adoption among patients with chronic noncancer pain and opioid use after intervention. In summary, we are proposing a complete stepped-wedge cluster-randomized design with 5 time periods (including baseline), with each period being 3 months, and 2 clinics switching from control to intervention at each of the 4 steps (for a total study duration of 15 months). We expect an average of ~10 qualifying patients being seen per clinic per quarter. Based on preliminary data from an ongoing study by our team [60], CDS without tailored implementation support is being used in 8% of visits involving patients with chronic pain. Therefore, assuming a pre-intervention CDS adoption rate of 5% among study clinics, we would have 90% power (*α*=0.05) to detect a difference between adoption rates of 9.1% or greater (i.e., 5% vs. 14.1%), for intraclass correlation (ICC) values among patients seen by the same provider up to 0.25.

#### Analysis

Descriptive statistics will be summarized for baseline survey measures overall and by clinic pairs that “step” into the intervention. Any observed baseline differences among randomized pairs will be accounted for as fixed effects in the primary analysis. To compare the effect of the intervention with usual practice on CDS outcome measures in a stepped-wedge design, we will utilize generalized linear mixed models (binomial distribution, logit link) with random effects for clinic. This model will incorporate independent variables (fixed effects), consider the general time trend, and allow for the intervention effect to grow over time. We will estimate the intervention effect with the within-site difference between CDS documentation rates pre- and post-intervention, averaging across practices and accounting for possible secular trends which might confound results. From the perspective of the patients (and their encounters) that will contribute data, this trial will be an open-cohort design, as it is possible that multiple encounters from patients will be included in the ultimate analysis [53]. If we observe repeated measures at the patient level, we will include a random effect of patient. We will also utilize this modeling framework to assess whether patient age, sex, and/or race/ethnicity are associated with the intervention effect via statistical interactions of the relevant fixed effects. As our statistical tests are specified a priori and our proposed outcome measures are highly related, we will report *p* values (and 95% confidence intervals) rather than adjust for multiple comparisons [61, 62]. We will use sequential mixed methods [63] to identify the multilevel determinants of implementation and sustainability according to EPIS [34] and RE-AIM [45].

Given the small sample of clinics and providers, we will use descriptive statistics to summarize the quantitative data from the pre- and post-trial surveys to assess provider- and clinic-level measures. Qualitative data collected from in-depth interviews with clinicians will provide additional context to clinician survey responses. Interview transcripts will initially be reviewed using framework analysis for rapid and multi-disciplinary assessment of key findings [64]. We will use a thematic content analysis approach to analyze interview data, given the structured nature of our inquiry into the specific domains surrounding implementation outcomes [65]. Qualitative analyses will involve data coding, within-clinic analysis, and cross-clinic analysis. A descriptive clinic-by-theme matrix template will be developed with primary thematic categories based on the interview questions [66]. We will independently use the matrix template to abstract findings and completed matrices will be reviewed in team meetings to resolve discrepancies, establish inter-rater reliability, and inform the development of a codebook for in-depth analysis. The qualitative analysis will be conducted by trained coders using iterative stages of deductive and inductive coding, enabling identification and description of emerging themes [63]. Selective member checking will be conducted to enhance validity.

### Approach for aim 3

#### Measures

1. Shared decision making: We will assess the receipt of shared decision making as reported by the patient using the 9-item Shared Decision Making Questionnaire (SDM-Q-9) instrument [67]. Items are scored on a 6-point Likert scale, ranging from 0 (“strongly disagree”) to 5 (“strongly agree”). An SDM-Q-9 sum score (ranging from 0 to 100) is calculated with higher values indicating a higher extent of shared decision making.
2. Pain and function: As a secondary outcome, effectiveness will be assessed via phone follow-up with qualifying patients. Pain and function will be assessed among all patients using PEG-3 at their index visit [68]. The 3-item scale measures pain on average, its interference with enjoyment of life, and with general activity, scored on 10-item scales, then divided by 3 to get a mean score (out of 10) on overall impact.
3. Pain treatment patterns: Because the primary intent of PainManager is to achieve optimal care based on shared decision making around chronic pain management, we will measure several indicators of pain treatment risks. These outcomes represent clinical process measures that are linked to opioid-related patient safety risks. Via EHR data extracted for qualifying patients during the study period, we will construct binary measures of treatment choices that increase opioid-related risks as defined by CDC guidelines [69]: (i) any opioid prescription, (ii) opioid prescriptions ≥50 MME/day, (iii) opioid prescriptions ≥90 MME/day, and (iv) benzodiazepine prescription concurrent with opioid prescription. We will also construct binary measures of behaviors recommended by the CDC guidelines to decrease opioid-related risks: (i) prescriptions for non-opioid pain medications, (ii) prescriptions for non-pharmacologic pain treatments, (iii) urine drug screen orders, (iv) naloxone prescriptions, and (iv) prescription or referral for medication-assisted therapy (MAT). As the 2016 CDC opioid prescribing guideline is expected to be revised, we will update these outcomes for consistency as needed.
4. Maintenance: As measures of maintenance, we will assess pain and function, and pain treatment patterns at 1 and 3 , and 6 months post-visit. Although PEG-3 may be available in the EHR via PainManager, we also follow-up with patients at via telephone at 1 and 3 months post-visit for increased completeness of this outcome.

#### Sample size considerations

Statistical power considerations for Aim 3 focus on obtaining important feasibility data (rates of data collection, clinic/provider/patient adherence, etc.), as well as estimating variances and ICCs of effectiveness outcomes to be the primary outcomes of a larger trial (SDM-Q-9, PEG-3) [70, 71]. In this proposed stepped-wedge trial, we will attempt to collect patient-level SDM-Q-9 and PEG-3. We anticipate only collecting this measure on average from 50% of eligible patients (5 per clinic per quarter), leading to a final sample size of 25 per clinic over the 15-month period (200 total). This sample size will facilitate sufficiently precise estimates of feasibility to plan a future group randomized trial. To establish preliminary evidence of effectiveness to serve as the premise for a larger study, we will perform hypothesis testing with a higher significance level (0.20) [72]. In this scenario, we would have 80% power (*α*=0.20) to detect an effect size of 0.47 or greater, for intraclass correlation (ICC) values among patients seen by the same provider ranging between 0.05 and 0.20.

#### Analysis

We will use the same general analytic framework as described for Aim 2, with a focus on estimates (and 95% CIs) of feasibility outcomes and preliminary estimates of effectiveness with respect to shared decision making, pain, and function. We will also use generalized linear mixed models (Gaussian distribution, identity link) to test the effect of the intervention on these two primary Aim 3 outcomes, including a random effect for clinic and including time as a fixed effect to account for time trends. While we do not anticipate sufficient statistical power to definitively establish effectiveness as described above, we anticipate results from these models will establish precedent for a larger study and provide important data in planning the subsequent trial. As described in Aim 2, we can use this modeling framework for testing interactions of importance (sex, age, race/ethnicity). We will also do preliminary mediation analyses to model the hypothesized causal pathways of the effect of CDS (CDS➔SDM-Q-9➔PEG-3) that will inform subsequent larger studies.

#### Needs assessment and adaptation to non-Epic EHR sites

We will conduct a needs assessment and systems design workshop to preliminarily adapt PainManager for use in two additional OneFlorida clinics. We will work with CommHIT leadership to recruit a single healthcare organization in the OneFlorida network that has at least two clinics using a non-Epic certified EHR system. First, we will conduct a series of needs assessment calls with clinic administrators and representatives of the clinic IT support teams. EHR vendor representatives will be included as needed. These calls will focus on understanding local data structures and quality, EHR support for FHIR resources, and assessing technical needs for adapting PainManager to the clinics. Then, we will conduct a system design workshop similar to that employed in Aim 1. Attendees will include study team members, software development and IT personnel, and representatives of leadership, clinicians, and clinic staff from the two clinics. We will summarize to the group the functionality of the existing adapted PainManager CDS application used at UF Health. Next, through facilitated discussion and sketching exercises, participants will establish consensus on potential design adaptations to PainManager’s interface and functionality that would further enhance local usability and clinical usefulness. Software developers and clinic IT personnel participants will provide input on the feasibility of proposed changes.

## Discussion

This project will *demonstrate scalability* by extending PainManager, an existing CDS tool, beyond the developing institution to new sites with different end users and workflows. The project will *promote interoperability* by using Health Level 7 (HL7) FHIR and Clinical Quality Language (CQL) to facilitate consistent and replicable implementation of the CDS across different healthcare systems. The work will also *incorporate patients’ perspective* through a series of patient interviews and design sessions that adapt and tailor the existing CDS to patient goals and preferences. Finally, this project will *contribute to a collaborative community of CDS developers and implementers* by participating in the CDS Connect Work Group and collaborating with other grantees on scientific and practice-oriented publications and presentations.

In the proposed study, we will implement CDS that disseminates evidence-based practice on the comparative effectiveness of opioids versus other treatments for chronic noncancer pain. Such evidence, which does not rule out opioid use, is reflected in the 2016 Centers for Disease Control and Prevention (CDC) Guideline for Prescribing Opioids for Chronic Pain [69]. The guideline recommends that non-opioids are preferred, but that opioids may be used when benefits outweigh risks. Despite the availability of many non-opioid treatment options, including non-pharmacologic options, clinicians and public health experts have raised concerns that the CDC guideline may have led to harms from abrupt opioid tapering, increased use of illicit opioids, and poor pain and function outcomes [73–76]. In some patients, opioids may be the best treatment option and are appropriate if prescribed consistent with clinical practice guidelines, including risk monitoring and mitigation. In summary, the best available evidence supports a careful, patient-centered weighing of risks and benefits when choosing among pain treatment options. Such a process can be facilitated by CDS, like PainManager.

This study will adapt and scale existing AHRQ-supported interoperable CDS for patient-centered chronic pain care. The research will generate evidence on strategies for implementing interoperable CDS in new clinical settings across different types of EHRs. The study will also inform tailored implementation strategies to be further tested in a subsequent hybrid effectiveness-implementation trial. Together, these efforts will lead to important new technology and evidence that patients, clinicians, and health systems can use to improve care for millions of Americans who suffer from pain and other chronic conditions.

## Supplementary Information

**Additional file 1.** Standards for Reporting Implementation Studies: the StaRI checklist for completion.

## Data Availability

No study data have been collected yet. Upon study completion, any datasets used and/or analyzed during the current study will be available from the corresponding author (CAH) on reasonable request.

## Abbreviations

AHRQ: Agency for Healthcare Research and Quality
API: Application programming interface
CDS: Clinical decision support
CQL: Clinical Quality Language
EHR: Electronic health record
EPIS: Exploration, Preparation, Implementation, Sustainment
FAIR: Findable, Accessible, Interoperable, and Re-usable
FHIR: Fast Healthcare Interoperability Resources
HL7: Health level 7
IT: Information technology
ORIC: Organizational readiness for implementing change
PCOR: Patient-Centered Outcomes Research
PCORI: Patient-Centered Outcomes Research Institute
RE-AIM: Reach, Effectiveness, Adoption, Implementation, Maintenance
SDM-Q-9: 9-Item Shared Decision Making Questionnaire
SMART: Substitutable Medical Applications, Reusable Technologies
SUS: System usability scale
